# *Notes from the Field*: Complications of Mumps During a University Outbreak Among Students Who Had Received 2 Doses of Measles-Mumps-Rubella Vaccine — Iowa, July 2015–May 2016

**DOI:** 10.15585/mmwr.mm6614a4

**Published:** 2017-04-14

**Authors:** Matthew Donahue, Allison Schneider, Ugochi Ukegbu, Minesh Shah, Jacob Riley, Andrew Weigel, Lisa James, Kathleen Wittich, Patricia Quinlisk, Cristina Cardemil

**Affiliations:** ^1^University of Iowa Carver College of Medicine; ^2^Duke University School of Medicine, Durham, North Carolina; ^3^Epidemic Intelligence Service, CDC; ^4^Johnson County Public Health Department, Iowa; ^5^University of Iowa Student Health & Wellness; ^6^Iowa Department of Public Health; ^7^Division of Viral Diseases, National Center for Immunization and Respiratory Diseases, CDC.

During July 2015–May 2016, a mumps outbreak occurred at the University of Iowa, which is located in Johnson County (*1*). A total of 301 cases of mumps were diagnosed among students. To characterize the outbreak, the Johnson County Public Health Department, the Iowa Department of Public Health, and the University of Iowa, with assistance from CDC, conducted an investigation through telephone interviews, medical chart abstractions, and review of immunization records. Among 287 (95%) students with mumps for whom clinical information was available, 20 (7%) patients with complications were identified (16 self-reported and four clinician-diagnosed). The 20 cases included 15 (5%) cases of orchitis, three (1%) of transient hearing loss, two of mastitis, and one of meningitis (one patient had both orchitis and transient hearing loss). All 20 patients had documentation of receipt of at least 2 doses of measles-mumps-rubella vaccine. Because data are limited regarding the presentation and clinical course of mumps complications in persons who have received 2 doses of mumps-containing vaccine, three illustrative cases of complications (orchitis, transient hearing loss, and meningitis) in students with mumps are presented.

## Patient A

On November 17, 2015, a man aged 21 years developed right jaw pain and swelling and received a clinical diagnosis of mumps parotitis; the diagnosis occurred 2 weeks after his roommate had received a mumps diagnosis. By the ninth day after symptom onset, the patient’s parotitis had resolved, but he reported a fever of 101.0°F (38.8°C), and 2 days later, he developed left testicular pain and swelling. Orchitis was diagnosed and he was prescribed nonsteroidal anti-inflammatory drugs and ice packs and had no further follow-up care.

## Patient B

On October 13, 2015, a woman aged 21 years developed progressive right ear pain, cough, and shortness of breath. Two days later, she was treated in a hospital emergency department where she received a diagnosis of right otitis externa and left otitis media, for which she was prescribed amoxicillin and analgesics. Later that day, she went to the University of Iowa Student Health Center because of worsening respiratory symptoms. During that encounter, she was also noted to have right bullous myringitis (purulent inflammation of the tympanic membrane), right parotitis suspected to be mumps, and suspected pneumonia. Azithromycin was prescribed empirically to treat both the bullous myringitis and atypical pulmonary pathogens. A polymerase chain reaction (PCR) test for mumps was performed on a buccal swab specimen and was negative. However, her symptoms and epidemiologic link to the outbreak met the Council of State and Territorial Epidemiologists case definition for a probable case of mumps. One day later, she noticed tinnitus and diminished hearing in her right ear; on day 8, she had audiology testing and was evaluated by an otolaryngologist, at which time she received a diagnosis of moderate right sensorineural hearing loss, attributed to mumps, and conductive hearing loss, attributed to otitis media and myringitis. She was treated for 1 week with prednisone, and all her symptoms resolved by the thirteenth day after onset of parotitis. No repeat audiology testing was performed.

## Patient C

On November 2, 2015, a man aged 21 years developed left facial pain and swelling and tested positive for mumps by PCR on a buccal swab specimen. Twenty-two days after onset of symptoms, he was treated at an emergency department for neck stiffness, fever, and tachycardia. A lumbar puncture was performed, and he was empirically treated for meningitis with acyclovir and ceftriaxone. Volume of cerebrospinal fluid was inadequate for performing PCR testing for mumps, but Gram stain and bacterial culture were negative, and analysis was consistent with viral meningitis (40 lymphocytes/mm^3^, 60 mg/dL of protein, and 67 mg/dL of glucose). Because the onset of mumps-related meningitis has been described as ranging from 4 days before the onset of parotitis until 3 weeks after (*2*), the patient’s viral meningitis diagnosis was attributed to mumps. He was discharged with recommendations for symptomatic care, and meningeal symptoms resolved within 1 week.

Complications of mumps have been reported less frequently since licensure and widespread use of mumps-containing vaccines. However, this case series demonstrates that complications still occur, even in persons who have received the recommended 2 doses of measles-mumps-rubella vaccine. In addition, complications can occur at varying times throughout the course of the illness and in the absence of parotitis (*2*,*3*). Health officials should remain vigilant for these complications and their relation to mumps, and when mumps is suspected, conduct PCR testing on a buccal swab specimen and serology on a serum specimen (*4*,*5*).
